# Cell-Free DNA Methylation: The New Frontiers of Pancreatic Cancer Biomarkers’ Discovery

**DOI:** 10.3390/genes11010014

**Published:** 2019-12-23

**Authors:** Mariarita Brancaccio, Francesco Natale, Geppino Falco, Tiziana Angrisano

**Affiliations:** 1 Department of Biology and Evolution of Marine Organisms, Stazione Zoologica Anton Dohrn, Villa Comunale, 80121 Naples, Italy; 2 Department of Biology, University of Naples Federico II, 80126 Naples, Italy; 3 Biogem Scarl, Istituto di Ricerche Genetiche “Gaetano Salvatore”, Via Camporeale, 83031 Ariano Irpino, Italy

**Keywords:** biomarker, cell-free DNA, chronic pancreatitis, DNA methylation, early diagnosis, epigenetic signature, pancreatic cancer, pre-neoplastic, prognosis

## Abstract

Pancreatic ductal adenocarcinoma (PDAC) is among the most lethal cancer types world-wide. Its high mortality is related to the difficulty in the diagnosis, which often occurs when the disease is already advanced. As of today, no early diagnostic tests are available, while only a limited number of prognostic tests have reached clinical practice. The main reason is the lack of reliable biomarkers that are able to capture the early development or the progression of the disease. Hence, the discovery of biomarkers for early diagnosis or prognosis of PDAC remains, de facto, an unmet need. An increasing number of studies has shown that cell-free DNA (cfDNA) methylation analysis represents a promising non-invasive approach for the discovery of biomarkers with diagnostic or prognostic potential. In particular, cfDNA methylation could be utilized for the identification of disease-specific signatures in pre-neoplastic lesions or chronic pancreatitis (CP), representing a sensitive and non-invasive method of early diagnosis of PDAC. In this review, we will discuss the advantages and pitfalls of cfDNA methylation studies. Further, we will present the current advances in the discovery of pancreatic cancer biomarkers with early diagnostic or prognostic potential, focusing on pancreas-specific (e.g., *CUX2* or *REG1A*) or abnormal (e.g., *ADAMTS1* or *BNC1*) cfDNA methylation signatures in high risk pre-neoplastic conditions and PDAC.

## 1. Introduction

CP is a long-lasting inflammation of the pancreas that alters the normal structure and function of the organ, and it represents one of the major risk factors for the development of PDAC [1]. PDAC is the third leading cause of global cancer related mortality, with a five year survival rate of only ≈9% for patients receiving treatment [2,3]. PDAC is an aggressive disease whose symptoms are very similar to those of CP, and it usually is at an advanced stage at the time of diagnosis (>75% of diagnosed cases are stage III/IV diseases) [4]. As of today, surgical resection, when applicable, is the only a potentially curative treatment, and novel biomarkers are clearly needed to identify subsets of patients likely to benefit from chemoradiotherapy [5,6].

Nonetheless, the progression from pre-neoplastic lesions to PDAC is slow [7], and the lag period between the diagnosis of CP and overt tumor is usually one or two decades [1]. This long latency period offers a strategic opportunity for early diagnosis and, thus, curative treatment, provided that biomarkers with robust predictive power are discovered [8]. The only FDA approved biomarker for prognostic surveillance of patients with known PDAC is carbohydrate antigen 19-9 (CA19-9 or sialylated Lewis antigen). However, this antigen displays low sensitivity and specificity for the detection of the disease [9], and its diagnostic utilization is discouraged unless in combination with other circulating biomarkers [10]. The analysis of pancreatic juice is more informative and allows gathering a substantial fraction of the current knowledge [11,12]. Yet, the specimen collection, when utilizing this method, is cumbersome and invasive.

The lack of non-invasive diagnostic methods to detect precancerous lesions in high risk subjects has driven the scientific community to explore new strategies. The analysis of DNA methylation changes in circulating cfDNA has proven a worthy approach.

DNA methylation is the DNA methyltransferases (DNMTs) mediated covalent addition of a methyl group to the carbon 5 of cytosines, resulting in 5-methylcytosine (5mC). This chemical reaction mainly occurs at CpG dinucleotides. In most cases, when methylated CpGs are found at the regulatory regions of nearby genes, transcription of such genes is suppressed [13]. On the contrary, ten-eleven translocation (TET) protein mediated 5mC oxidation ultimately results in DNA demethylation, which is generally associated with transcriptional activation [14]. DNA methylation plays an important role in the development and progression of cancer, altering the chromatin structure and ultimately leading to silencing of tumor suppressor genes or activation of oncogenes [15]. Changes in DNA methylation often occur at early stages of tumorigenesis [15]. This has been confirmed for bladder [16], breast [17], colorectal [18], and lung cancers [19,20], as well as for pre-neoplastic lesions of the pancreas [21,22,23,24].

In the past decade, great efforts have been made in investigating methylation signatures in circulating cfDNA. cfDNA carries methylation markers that enable the identification of tissue-specific cell death [25,26] and are more broadly informative, sensitive, and specific than individual DNA mutations [11,27]. Further, sample collection is minimally invasive and allows adequate follow-up under negligible stress conditions for the patient.

Indeed, the FDA has recently approved the use of methylation biomarkers for colorectal cancer (Cologuard${}^{®}$, Exact Sciences Inc., Medison, USA; EpiproColon${}^{®}$, Epigenomics Gmbh, Berlin, Germany) and bladder cancer (Exact Sciences Inc., Madison, USA). However, no tests for the early diagnosis of PDAC are currently available. Exploiting the relation between abnormally methylated cfDNA and pre-neoplastic lesions of the pancreas or CP may become a game changing approach for the development of PDAC-specific diagnostic or prognostic tools. In this review, we will discuss the advantages and pitfalls of cfDNA methylation studies. Further, we will describe abnormal cfDNA methylation signatures in high risk pre-neoplastic conditions and PDAC and present the current advances in pancreatic cancer diagnostic and prognostic biomarker discovery.

## 2. Genetic and Epigenetic Implications of cfDNA in Cancer Diagnosis

cfDNA is composed of short double stranded segments of nucleic acids that are not associated with cells or cell fragments. cfDNA is present in the plasma and serum as polynucleotide chains of 0.1–20 kilobase-pairs [28,29]. Trauma [30], stroke [31], and even strenuous exercise [32] can induce a significant increase of cfDNA plasma concentration. In healthy individuals, cfDNA concentration ranges from 0 to 100 ng/mL of blood [33]. In comparison, cancer patients present on average four to 40 times greater levels, with an upper range exceeding 1000 ng/mL [34,35,36,37,38,39]. The higher cellular turnover observed in cancer would explain at least in part the higher plasmatic cfDNA levels observed. For example, in colorectal cancer, an estimated 3.3% of tumor DNA is released into the circulation, daily [40]. Since all tumors exhibit genetic alterations, including single base substitutions, insertions, or translocations, this genetic information becomes immediately available when cfDNA is sequenced. Hence, sequence analysis of plasma cfDNA has quickly become a non-invasive method to assess the presence of the disease in cancer patients, propelling the research in this field as documented by >5500 studies since 2010.

### 2.1. Pitfalls in cfDNA Data Interpretation

The interpretation of circulating cfDNA sequence data is not without hurdles. For PDAC, mutations in the circulating DNA of the *KRAS* gene were uncovered already in the early 1990s [41], and improvement of these methods was attained in the past years [42,43,44]. Circulating mutated *KRAS* is found in 26–73% of the subjects affected by PDAC, and it is associated with a poor prognosis [45]. These figures are in contrast with the reports of primary PDAC biopsies, with *KRAS* mutation frequency nearing 100% for PDAC [46]. Such discrepancy may underlie both technological and biological limitations. For the former, the optimized collection of the scarcely abundant material, the decrease of the limit of detection, or further refinement of the analysis algorithms would be desirable improvements, and efforts have been made in this direction [21,47]. For the latter, it may be possible that a fraction of PDACs do not release mutant *KRAS* cfDNA in the bloodstream [43].

Indeed, it is accepted that cfDNA can be actively released by viable cells or passively released by apoptotic or necrotic cells. These cells can be either healthy or neoplastic. The latter can also be found in the bloodstream as circulating tumor cells (CTCs) [38,48], which may contribute to the global levels of circulating cfDNA. However, the existence of cfDNA in the absence of CTCs is equally possible, suggesting that the two biomarkers may have different origins [44].

One factor adding to the complexity of this scenario is the high genetic heterogeneity observed in cancer. Often times, two tumors of the same type share just a few somatic “driver” mutations that promote the disease [49]. This has been ascertained for a large variety of tumors in both primary and liquid (i.e., circulating cfDNA) biopsies [43,49,50]. The remainder of the genetic alterations, spanning from tens to thousands depending on the tumor type, are “passenger” mutations, conferring no selective growth advantage. For pancreatic tumors, the overall somatic mutation frequency spans about 0.1–3/Mbp [49], and the circulating mutated fragments are in the order of 0.5–1500/mL [44].

Together, the combination of the diverse origin, heterogeneous genetic background, and interindividual variability of plasmatic levels limit the potential information provided by the genetic sequence alone and make the interpretation of circulating cfDNA sequence data a complex task.

Thus, although driver mutations in *KRAS* have prognostic value for PDAC, they currently cannot be used to detect the early emergence of the disease [42].

### 2.2. Exploiting DNA Methylation Signatures for the Identification of Circulating cfDNA

DNA methylation profiles in both malignant and non-malignant conditions appear to be very similar if they originate from the same tissue [25,26,51]. For example, Sun and colleagues investigated circulating cfDNA of pregnant women, transplanted subjects, or individuals affected by hepatocellular carcinoma [26]. By utilizing large tissue-specific DNA methylation fingerprints as the baseline [52], they were able to deconvolve the heterogeneous signals collected from the circulating pool of cfDNA and to pinpoint the fraction of molecules originating from a given tissue. Similarly, Lehmann-Werman and coworkers developed a method of detecting specific tissue cell death [25]. They interrogated tissue methylome databases in order to identify DNA methylation patterns that were specific for given cell types or tissues. Then, they sought to identify cell/tissue-specific DNA methylation patterns in the plasma of individuals presenting diverse conditions, by probing their cfDNA. With this approach, they were able to identify the DNA of oligodendrocytes precisely in patients with relapsing multiple sclerosis, pancreatic β cells’ DNA in the bloodstream of patients with type 1 diabetes or newly diagnosed islet transplant recipients, or exocrine pancreatic DNA in patients with PDAC or CP.

In addition to DNA methylation, cfDNA hydroxymethylation (5hmC) profiles may prove useful for the identification of tissue- or disease-specific signatures in the circulating pool of cfDNA. For example, Li and colleagues analyzed cfDNA 5hmC profiles in patients diagnosed with colorectal, gastric, liver, thyroid, or pancreatic cancer, using genomic DNA of paired tumors or adjacent tissues as the comparator [53]. They found robust cancer associated cfDNA 5hmC signatures that were characteristic for specific cancer types. For colorectal and gastric cancer, these signatures were highly predictive of the disease, superior to conventional biomarkers, and comparable to 5hmC profiles obtained from primary biopsies.

Thus, the integration of genetic sequence data with epigenetic information appears to be critical for the deconvolution of the highly heterogeneous circulating cfDNA pool. This breakthrough will have a major impact on the interpretation of cfDNA sequence data, allowing researchers to focus on disease-specific subsets of molecules and to stratify the heterogeneous genetic background in an unbiased fashion. Applications may be diverse, including the investigation of the intratumoral heterogeneity, the reconstruction of somatic rearrangements, the resistance of the tumor to possible drug therapies, or the assessment of the minimal residual disease.

## 3. cfDNA Methylation Biomarkers in Pancreatic Cancer or Pancreatic Pre-Neoplastic Conditions

In the past two decades, an extensive body of evidence describing aberrant DNA methylation of >100 genes has been collected from pancreatic juice or primary biopsies of PDAC or CP [8]. While some of these targets were validated in independent cohorts, results are still controversial for the majority of these genes. More importantly, to develop a robust non-invasive test, DNA methylation signatures from primary tissue require validation in circulating cfDNA. To date, a limited number of studies investigating cfDNA methylation in PDAC have been conducted. A literature search for relevant studies using the search terms “circulating cell-free DNA” OR “circulating tumor DNA” AND pancreatic cancer conducted on the PubMed database resulted in 187 studies since 2010. Of these, only 21 (11%) investigated cfDNA methylation. Typically, the standard design of these studies hinges on the following elements:

Comparator: Results obtained from PDAC are typically compared to healthy subjects or CP. When available, other pre-neoplastic pancreatic lesions such as pancreatic intra-epithelial neoplasia (PINs) or intramucinous pancreatic neoplasia (IMPNs) are included.

Cohort design: For a desirable approach, a pre-set minimum number of subjects satisfying pre-defined inclusion or exclusion criteria in each cohort of the study is expected. This would ensure sufficient power for the analysis and the achievement of robust conclusions. However, depending on the criteria adopted and on the rarity of the condition, the numerosity of the cohorts is often a limiting factor, especially for prospective studies.

Methodological approach. A large number of methods enabling DNA methylation assessment exist. The majority of these methods rely on the relatively fast bisulfite mediated deamination of un-methylated cytosines into uracils. DNA methylation differences can then be assessed, quantitatively or non-quantitatively, by either targeted amplification of regions of interest or, on a broader scale, by means of microarray or next-generation sequencing (NGS) of the whole genome. The advantages or disadvantages of the methods utilized in the studies discussed in this review are summarized in Table 1. Methylation-specific PCR (MSP) is among the most commonly utilized methods, being inexpensive and able to produce fast results. However, this method is not devoid of downsides, most importantly the lack of standardization.

Data analysis: As discussed earlier, a variety of biological or methodological pitfalls may render the analysis of cfDNA methylation data a complex task. For example, to draw a conclusion about as to whether a given biomarker is a predictor of the disease or has prognostic value, it may be necessary to interrogate many variables simultaneously. Depending on the relation between the interrogated variables, a variety of statistical models (e.g., naive Bayes classifiers, multivariate logistic regression) have increasingly been utilized to tackle the complex biology or alleviate the confounding effect of dependent variables [54,55,56]. Typically, a subset of the data is used to train the model to detect the desired targets. The remainder of the samples are then used to actually test the biomarkers.

The studies discussed in this review investigated either the abnormal decrease or lack of DNA methylation (hypomethylation), or the abnormal increase of DNA methylation (hypermethylation), or both, as the coexistence of hypermethylation and hypomethylation events is equally possible.

### 3.1. cfDNA Hypomethylated or Non-Methylated Targets

One of the first studies pioneering the cfDNA methylation based detection of PDAC was conducted by Levenson’s group [56]. cfDNA methylation profiles of 56 gene promoters from treatment naive patients suffering from PDAC were compared to profiles obtained from healthy volunteers without evidence of pancreatic disease. Stage I–IV, resectable, and non-resectable cases, and controls were age matched. The assay utilized in this study relied on methylation sensitive endonucleases and a microarray mediated differential methylation hybridization approach (MethDet-M^3^; Table 1) [57]. The most informative genes (*CCND2*, *PLAU*, *SOCS1*, *THBS*, and *VHL*) were included in a composite biomarker. The key feature of this study was the assessment of hypomethylation in tumor specimens compared to controls. In this method, only the non-methylated status of a promoter could be unequivocally assigned to tumor cells in the heterogeneous specimens. The authors underlined how unmethylated genes were more informative than methylated genes because the latter can originate in any of multiple components of a heterogeneous sample. Conversely, a lack of methylation can be attributed to all components. The assay distinguished healthy pancreas from PDACs with a sensitivity and a specificity of 76% and 59%, respectively. Because this test assesses the absence of DNA methylation, the comparison with techniques that detect cancer related hypermethylation is difficult, and these findings remain controversial. Nonetheless, the authors addressed two of the pitfalls in the field of cfDNA biomarker discovery, that is the heterogeneity of the sample as a major cause of the confounding effect and the limited probing capacity as the main source of non-reproducibility.

The intuition of deconvolving the heterogeneous signal of circulating cfDNA with tissue-specific baseline information [26] allowed Lehmann-Werman and coworkers to identify DNA methylation patterns specific for exocrine pancreas, even if the latter was diluted 1:1000 in lymphocyte DNA [25]. Similar to MethDet-M^3^, this method hinges on the absence of DNA methylation in the region of interest. Specifically, two regions belonging to the genes *CUX2* and *REG1A* were unmethylated in exocrine pancreas and methylated in most other tissues, including endocrine pancreas. Hence, the identification of circulating unmethylated copies of cfDNA from these regions would be indicative of release from exocrine pancreas. Based on this observation, the authors probed the plasma of subjects suffering from PDAC. Interestingly, the assay enabled the identification of an exocrine pancreas-specific signal in stage I disease, suggesting that it has the potential to identify cell death in PDAC at a resectable stage. Furthermore, analysis of plasma from subjects suffering from CP revealed exocrine pancreas-specific cfDNA levels comparable to PDAC. Nonetheless, patients with CP showed a clearer signal with a marker that was unmethylated in both acinar and ductal cells (*REG1A*), whereas patients with PDAC had a stronger signal with a marker that was preferentially unmethylated in ductal cells (*CUX2*), perhaps reflecting the different epigenetic identities of dying cells in the two pathologies (Table 2).

Advancements in this direction are desirable, as this approach underlines a pivotal role of DNA methylation signatures as potent labels of the disease-specific circulating cfDNA pool.

### 3.2. cfDNA Hypermethylated Targets

The majority of the studies investigating cfDNA methylation in PDAC or pancreatic pre-neoplastic conditions focused on DNA hypermethylation.

Park and coworkers investigated the DNA methylation levels of six genes (*CDKN2A*, *NPTX2*, *PENK*, *SFRP1*, *RASSF1A*, and *UCHL1*) in the plasma of individuals suffering from unresectable advanced or metastatic PDAC and CP [58]. The probed genes had previously been reported to present high DNA methylation in primary PDAC [12,59]. In this study, bisulfite treated cfDNA was amplified by MSP with primers containing at least four CpG sites, to increase specificity (Table 1). Direct sequencing of the amplicons was utilized to quantify the methylation status of each CpG site included in the amplicon. DNA methylation was detected in 81% of PDACs (13 of 16 cases) and 61% of CP (eight of 13 cases), with at least one gene affected in either condition. In contrast, less than 4% of healthy pancreas (one of 29) presented DNA methylation. The most frequently methylated gene was *NPTX2* (38% and 31% for PDAC and CP, respectively), while the least methylated gene was *RASSF1A* (6% and 8% for PDAC and CP, respectively). However, the high interindividual variability (e.g., smoking or treatment history) and the small cohort size limited the power of the analysis, and significant differences between PDAC and CP could not be confirmed, except for *CDKN2A*, which was specifically methylated in PDAC, but not in CP. *NPTX2* hypermethylation status was confirmed in a larger cohort by the same authors [60]. Circulating cfDNA from 104 treatment naive patients affected by PDAC at different stages was examined. *NPTX2* methylation levels were significantly higher in PDAC, compared to CP or benign biliary stone disease (sensitivity and specificity were 80% and 76%, respectively) and were positively correlated with disease stage.

More information on the cfDNA methylation state of three of these genes was provided by a recent preliminary work from Singh and colleagues [61]. The authors assessed by QMSP (Table 1) the absolute copy number of methylated or unmethylated *NPTX2*, *PENK*, *SPARC*, and *UCHL1* cfDNA in individuals suffering from PDAC or CP, compared to healthy subjects. Quantitative DNA methylation levels were expressed as methylation indices (MIs), that is the fraction of the methylated copy number over the total detectable cfDNA. MIs were utilized to compare gene methylation levels in different groups and to correlate with clinicopathological features and survival of PDAC patients. Higher MIs for all four genes were found in PDAC patients compared to healthy patients. Higher MIs for *NPTX2* and *SPARC* were able to distinguish PDAC from CP, confirming previous observations [58,60]. Further, *SPARC* was associated with stage IV, metastasized disease, and poor survival, while *UCHL1* was found to correlate with the stage of the disease. The authors suggested that a combined utilization of *NPTX2*, *SPARC*, and *UCHL1* may provide a valuable diagnostic and prognostic tool, distinguishing CP from PDAC patients, and estimating the survival of the latter. Although quantitative, this study was conducted in small cohorts, and validation in larger independent cohorts is highly required to substantiate the results.

In a follow-up work from Levenson’s group, cfDNA methylation in treatment-naive subjects suffering from PDAC, CP, or healthy controls was compared by means of the MethDet-M^3^ method (Table 1) [55]. The study was designed to capture differences of ≥30% in cfDNA methylation proportions between the examined groups with a significance level of 5%. Seventeen gene promoters were identified as differentially methylated. These were further utilized to create disease-specific classifiers, that is combinations of genes that were specific for the detection of a given condition (e.g., PDAC or CP). Eight genes (*BRCA1*, *CCND2*, *CDKN1C*, *MLH1*, proximal and distal *PGR* promoter regions, *SYK*, and *VHL*) were useful to distinguish healthy pancreas from CP, with a sensitivity and a specificity of 82% and 78%, respectively. For all genes in this panel, promoter DNA methylation was more frequent in individuals with CP, with *CCND2*, *CDKN1C*, and the proximal *PGR* promoter region being methylated in >75% of CP subjects as opposed to about 15% in healthy individuals. Fourteen genes (*CCND2*, *CDKN1C*, *CDKN2B*, *DAPK1*, promoter A of *ESR1*, *MGMT*, *MLH1*, *MUC2*, *MYOD1*, *PGK1*, the proximal region of the *PGR* promoter, *RARB*, *RB1*, and *SYK*) were able to distinguish CP from PDAC with a sensitivity and specificity of 91%. Interestingly, although some genes were included in both classifiers groups (i.e., healthy vs. CP and CP vs. PDAC), all genes that were hypermethylated in CP displayed hypomethylation in PDAC (*CCND2*, *CDKN1C*, *MLH1*, the proximal region of the *PGR* promoter, and *SYK*) (Table 2). As CP often precedes PDAC, dynamic DNA methylation patterns for a given set of genes may underlie the progression of the disease. Although well designed, this study utilized small cohorts (*n* = 30 for each group), and validation in larger independent cohorts is highly desirable to confirm these findings.

**Table 2:** Summary of the major findings discussed in this review: (top) genes utilized for the heterogeneity deconvolution of circulating cfDNA and identification of pancreatic cfDNA (from [25]); (bottom) relevant biomarkers for PDAC detection at different stages (from [21,55,62]).

Genes Useful to Identify Circulating Exocrine Pancreatic cfDNA						
***CUX2***	Ductal cell marker			Increased signal in PDAC		
***REG1A***	Ductal and acing cell marker			Increasing signal in CP		
**Frequency of cfDNA methylation biomarkers (%)**						
Gene	healthy	CP	PIN	PDAC I	PDAC II	PDAC III/IV
***ADAMTS1***	<3	-	25	25–88	78–90	55
***BNC1***	3–7	5	70	63–95	56–95	100
***CCND2***	17	82	-	-	-	24
***CDKN1C***	60	90	-	-	-	27
***MLH1***	22	78	-	-	-	27
***PGR (prox)***	14	76	-	-	-	37
***SYK***	57	89	-	-	-	57

*ADAMTS1* and *BNC1* have recently gained momentum as promising biomarkers for early diagnosis of PDAC. Yi and coworkers [21] designed a robust study starting from a transcriptomic based screening of pancreatic cell lines. From a pool of >1400 genes, a panel of eight genes was selected as it showed PDAC-specific DNA methylation signatures. Among these, *ADAMTS1* and *BNC1* showed a high methylation frequency in primary PDACs and in pre-neoplastic PINs (25% and 70% for *ADAMTS1* and *BNC1*, respectively) (Table 2). Little to no DNA methylation was observed in CP or normal pancreas. Importantly, because *BNC1* methylation was detected in PINs and PDACs while *ADAMTS1* methylation was more frequently observed in invasive PTs, the aggregate utilization of both genes would hold potential for early PDAC detection. To test these biomarkers, the authors probed the plasma of subjects suffering from Stage I–IV PDAC or healthy individuals from an independent cohort. An optimized “single tube” procedure was adopted to optimize the yield of the recovered material [47]. The sensitivity was determined based on the stringent assumption that all patients with PDACs would harbor *ADAMTS1* and *BNC1* gene methylation, while healthy subjects would not. Under these conditions, a sensitivity of 48% and 79% was estimated for *ADAMTS1* and *BNC1*, respectively. Specificity was 92% for *ADAMTS1* and 89% for *BNC1*. Combining the two genes slightly improved the sensitivity (81%), but worsened the specificity (85%). This aggregated biomarker presented promising predictive power, and its performance was confirmed by the same group in independent cohorts with improved results [62]. The authors examined cfDNA from subjects suffering from early stage PDAC and healthy controls. For the latter, history of drinking, smoking, or type II diabetes, known risk factors for pancreatic cancer, were reported. The performance of both genes taken individually was improved: methylation of *ADAMTS1* was found in 87.5% (7/8) of stage I, 77.8% (7/9) of stage IIA, and 90% (18/20) of stage IIB PDAC; similarly, *BNC1* was positive in 62.5% (5/8) of stage I patients, 55.6% (5/9) of stage IIA, and 65% (13/20) of patients with stage IIB disease. The aggregate biomarker showed positive cfDNA methylation in 100% (8/8) of stage I, 88.9% (8/9) of stage IIA, and 100% (20/20) of stage IIB disease, with a combined sensitivity of 97.3% and specificity of 91.6%. These results are encouraging, indicating that the combined cfDNA methylation status of *ADAMTS1* and *BNC1* is a powerful tool for detecting pancreatic cancer during the early stages (i.e., I and II) when curative resection of the tumor is still possible. Integration of CA19-9 levels did not improve the performance of the test, confirming that this antigen remains useful only in a subset of patients. Concomitant investigation of other predictive biomarkers may still help improve the cancer specificity and sensitivity of the test. For example, the presence of positive DNA methylation of *ADAMTS1* and *BNC1* together with mutated *KRAS* in individuals without overt disease would already identify a high risk population to be closely monitored by computed tomography or endoscopic screening.

DNA methylation based prediction models are powerful tools, and their utilization in diagnostics has grown rapidly. Henriksen and coworkers developed a diagnostic prediction model based on the DNA methylation levels of a panel of 28 genes [63]. The genes, including *BNC1*, *CDKN2A*, and *RASSF1A*, were selected based on previous literature findings. By means of an optimized accelerated bisulfite treatment protocol and MSP (Table 1), the authors probed the plasma of treatment naive subjects suffering from PDAC, CP, and acute pancreatitis (AP). A fourth group of subjects resulting in being screening negative for PDAC, but affected by benign pancreatic conditions were utilized as the control and included in the CP group. A statistically significant difference in the mean number of methylated genes was observed between PDAC (8.41) and the aggregate CP control group (4.46). Little to no DNA methylation was observed in AP subjects, and these observation were confirmed later in primary biopsies [64]. The DNA hypermethylation frequency was assessed for each gene in each group. To develop the prediction model, the authors utilized a logistic regression. Such a statistical model is able to model the probability of binary dependent variables. Therefore, the DNA methylation status of genes that showed a significant difference (20 of 28) between PDAC and the aggregate control group was treated as a binary variable (methylated/non-methylated). The authors included demographic or social parameters (e.g., age >65 years or smoking status) to improve the model performance. Then, the elimination of variables, one at a time (stepwise backward elimination) resulted in the optimal combination of variables presenting the highest predictive power. This process resulted in an aggregate biomarker composed of eight genes (*APC*, *BMP3*, *BNC1*, *MESTv2*, *RASSF1A*, *SFRP1*, *SFRP2*, and *TFPI2*) and a demographic parameter (age >65 years), with a sensitivity of 76% and a specificity of 83%. Despite the non-invasiveness and fast execution of this test thanks to a rapid deamination step, this study was exploratory, only relying on training data used to generate the prediction model. This would result in an overestimate of the test performance, and validation in an independent cohort is required to confirm the results.

Based on cfDNA methylation data obtained from the previously described cohort, Henriksen and colleagues focused on the development of prognostic biomarkers for PDAC [65]. The examined genes were the same as in Henriksen et al. [63]. Overall, patients presenting more than 10 hypermethylated genes had a hazards ratio (HR) of 2.03 (95% CI; 1.15-3.57) compared to patients with fewer methylated genes. By means of a multivariable Cox regression analysis and the stepwise backward elimination process [63], they developed survival prediction models. The final model included, *BNC1*, *GSTP1*, *SFRP1*, *SFRP2*, and *TFPI2* genes, and an ASA physical status score of three (that is, “severe systemic disease”). All genes yielded an HR greater than one, indicating that their methylation was associated with poor prognosis, except for *SFRP2*, whose methylation correlated with longer survival. The analysis of these genes provides interesting cues. However, independent validation in larger cohorts remains crucial to substantiate these findings.

The last study we discuss summarizes the most recent advances in the field, including the concept of methylation haplotype blocks (MHB) and machine learning algorithms. Briefly, adjacent CpG sites in mammalian genomes can be co-methylated due to the processivity of DNMTs or demethylases. Yet, discordant DNA methylation patterns exist and are found related to stochastic or uncoordinated molecular processes. CpG rich genomic regions exhibiting highly coordinated methylation are referred to as MHBs [66]. A large study from Liu and colleagues aimed to identify PDAC-specific DNA methylation signatures in subjects suffering from CP and PDAC [54]. The authors generated DNA methylation profiles from neoplastic and matched non-neoplastic pancreatic tissue and from cfDNA by means of RRBS (Table 1). Cross-comparison of primary biopsies, matched healthy tissue and circulating cfDNA methylation profiles, and quantification of DNA methylation status of MHBs resulted in the identification of >350 PDAC-specific DNA methylation signatures. These signatures were then utilized to build discriminative classifiers. Probing circulating cfDNA from patients suffering from PDAC, these classifiers were able to achieve an average sensitivity of 86% and average specificity of 88%. Preliminary data indicated that the key genes utilized to generate these classifiers functionally overlapped with genes that had previously been associated with PDAC, suggesting their implication in PDAC pathogenesis. This large database of PDAC-specific DNA methylation signatures holds potential for the identification of novel biomarkers, and it would serve as a reference for the validation of existing ones.

## 4. Discussion and Conclusions

In recent years, a variety of specimen types other than plasma (e.g., urine or sputum) have been probed for cfDNA based diagnostics [67,68,69]. Liquid biopsies are minimally invasive and allow adequate follow-up under negligible stress conditions for the patient, and their utilization in translational and clinical research is rapidly increasing. In this review, we described the current advances in the identification of early diagnostic or prognostic tools for PDAC based on liquid biopsies derived DNA methylation signatures.

The analysis of circulating cfDNA methylation is not without hurdles. A major limiting factor of the reproducibility of (cf)DNA methylation data is the lack of a common reporting standard for DNA methylation detection, both pertaining to methodological approaches and to the targets of the analysis. For the former, the popular, fast executable and cost containing MSP comes with drawbacks: the limited coverage of this method may result in different regions of the same target being probed. The decreasing cost of NGS technologies offers an appealing option for methodological standardization. As for the targets of the analysis, the coexistence of hypermethylated or hypomethylated DNA tracts would still result in confounding findings, if precise genomic coordinates are not defined. Thus, in light of the recent discovery of several candidate biomarkers, it would be highly desirable that efforts be put into the establishment of a common reporting standard.

Furthermore, besides the inherent complex biology of the disease, which is yet to be elucidated, the heterogeneity of the circulating pool of cfDNA molecules (i.e., different origins) and the high genetic variability associated with cancer (i.e., “passenger” vs. “driver” mutations) make the interpretation of the results difficult. This task has been facilitated by technological advancements that enable deconvolving the heterogeneity of circulating cfDNA and to pinpoint tissue- and disease-specific subsets of molecules, based on highly specific DNA methylation signatures [25,26,54]. This breakthrough will surely boost the utilization of liquid biopsies and, thus, the research in PDAC biomarker discovery. However, the downside of this technological advancement would be the decreased pool of “informative” molecules. This may become limiting especially in subjects presenting low plasmatic cfDNA concentration, as a decreased statistical power required to confirm the results would hamper the data analysis.

**Figure 1:**
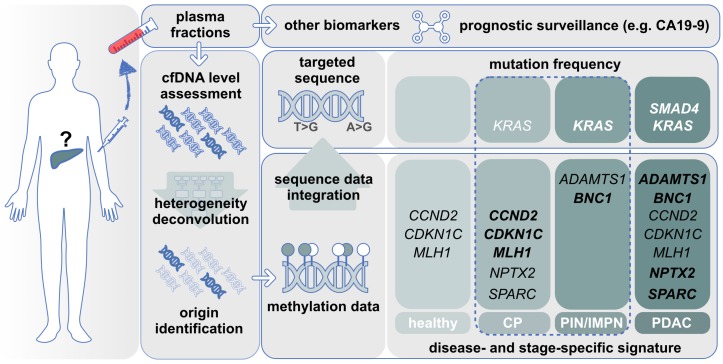
Cell-free DNA biomarker discovery for PDAC management. Tissue- and disease-specific DNA methylation signatures are identified in the circulating pool of cfDNA molecules. Integration of sequence data for disease-specific mutation would enhance the overall diagnostic and prognostic sensitivity. Gene names: high methylation level is indicated in bold. Dashed line: pre-neoplastic stage.

Most of the studies we described focused on genes undergoing aberrant hypermethylation during the pathogenesis of PDAC. For some of the genes, their methylation state may reflect different stages of the disease. For example, *BNC1* methylation is typically observed in PIN (70%) and all stages of PDAC (95%), while *ADAMTS1* methylation is seldom observed in PIN or stage I PDAC (25%) and more frequently in stage II PDAC (85%) (Figure 1) [21]. Hence, the integrated information obtained from the simultaneous DNA methylation status of these genes may capture the progression of the disease over time.

Indeed, great efforts have been made in the identification of highly predictive combinations of DNA methylation patterns, as it appears obvious that the individual contribution of single genes is not able to capture the complex biology of the disease. In this direction, the combination of multiple biomarker types may bolster the predictive power and enable early diagnosis. For example, there is evidence of salivary microRNA (miR) detection preceding systemic detection of cancer cell markers. Specifically, hsa-miR-23a and hsa-miR-23b are found upregulated in the saliva of subjects with pancreatic cancer precursor lesions (IMPN), whereas hsa-miR-210 and let-7c levels in saliva can distinguish CP from healthy subjects [69]. Similarly, serum miRs (miR-16-2-3p, miR-890, miR-3201, miR-602, and miR-877) enable the stratification of PDAC patients into low risk and high risk groups [70]. While detection and reproducibility issues still hinder the implementation of miRs in clinical practice, efforts in this direction have been made, and advances in this field in the near future are anticipated [71].

One aspect that is worth mentioning is the possibility for a given methylation signature of to be dynamic. In other words, depending on the stage of the disease, the same target region may show differences in its DNA methylation profile. This may be relevant in diseases such as PDAC where the inflammatory component plays a major role, whereby inflammatory stimuli may lead to aberrant DNA methylation homeostasis [72,73]. *CCND2*, *CDKN1C*, or *MLH1*, for example, are found hypermethylated in subjects suffering from CP [55]. The same genes in advanced PDAC presented DNA methylation frequencies comparable to healthy individuals [55]. Interestingly, *ADAMTS1* shows its highest methylation frequency in stage I/II PDAC [21,62], while in stages III and IV, a lower frequency is observed [21]. This line of evidence supports a dynamic methylation, and additional investigation aiming to correlate the DNA methylation levels with the disease stage is highly desirable.

In conclusion, DNA methylation signatures have great potential as molecular biomarkers to guide clinical management of PDAC, and the screening and diagnosis of the disease through circulating cfDNA will soon become available. The implementation of tissue- and disease-specific epigenetic biomarkers would represent an important paradigm shift in precision medicine, enabling early detection of the disease, serial assessment of its progression, follow-up during remission, or characterization of the treatment response or monitoring of the residual disease. To achieve this, more efforts are expected to be made in the standardization of reporting methods and in the validation of existing biomarkers.

## Figures and Tables

**Table 1:** Advantages and disadvantages of cfDNA methylation detection methods for biomarker discovery.

	Method				
**Features**	**MethDet-M^3^**	**MSP**	**QMSP**	**Targeted Amplicon Sequencing**	**RRBS**
**DNA methylation** **discrimination**	Methylation-sensitive endonuclease	Bisulfite Treatment	Bisulfite Treatment	Bisulfite Treatment	Bisulfite Treatment
**Coverage**	Medium/Low	Targeted	Targeted	Targeted	Medium
**Throughput**	Medium	Low	Medium	Medium/High	High
**Analytical** **Sensitivity**	Medium	Low	Medium	Low	Low
**Analytical** **Specificity**	High	Low/Medium	Medium	Medium	Medium
**Advantages**	Scalable (e.g., microarray)	Fast results Cost-effective	Quantitative	Scalable (e.g., NGS) Single CpG methylation status	Scalable (e.g., NGS) Single CpG methylation status
**Disadvantages**	Risk of false positive/negative	Limited coverage	Limited coverage	May require extensive data analysis	Extensive data analysis
**Popularity**	Unpopular	Very Popular	Popular	Popular	Popular

## Abbreviations

The following abbreviations are used in this manuscript:

AP	Acute pancreatitis
ASA	American Society of Anesthesiologists
cfDNA	Cell-free DNA
CP	Chronic pancreatitis
CTCs	Circulating tumor cells
DNMTs	DNA methyl transferases
HR	Hazard ratio
IMPN	Intramucinous pancreatic neoplasia
MHBs	Methylation haplotype blocks
MI	Methylation index
MSP	Methylation-specific PCR
NGS	Next-generation sequencing
PDAC	Pancreatic ductal adenocarcinoma
PIN	Pancreatic intra-epithelial neoplasia
RRBS	Reduced representation bisulfite sequencing
